# Interruption science as a research field: Towards a taxonomy of interruptions as a foundation for the field

**DOI:** 10.3389/fpsyg.2023.1043426

**Published:** 2023-03-22

**Authors:** Fabian J. Stangl, René Riedl

**Affiliations:** ^1^Digital Business Institute, School of Business and Management, University of Applied Sciences Upper Austria, Steyr, Austria; ^2^Institute of Business Informatics – Information Engineering, Johannes Kepler University Linz, Linz, Austria

**Keywords:** interruption, interruption science, interruption taxonomy, interruption classifications, interruption attributes, systematic literature review

## Abstract

Interruptions have become ubiquitous in both our personal and professional lives. Accordingly, research on interruptions has also increased steadily over time, and research published in various scientific disciplines has produced different perspectives, fundamental ideas, and conceptualizations of interruptions. However, the current state of research hampers a comprehensive overview of the concept of interruption, predominantly due to the fragmented nature of the existing literature. Reflecting on its genesis in the 1920s and the longstanding research on interruptions, along with recent technological, behavioral, and organizational developments, this paper provides a comprehensive interdisciplinary overview of the various attributes of an interruption, which facilitates the establishment of interruption science as an interdisciplinary research field in the scientific landscape. To obtain an overview of the different interruption attributes, we conducted a systematic literature review with the goal of classifying interruptions. The outcome of our research process is a taxonomy of interruptions, constituting an important foundation for the field. Based on the taxonomy, we also present possible avenues for future research.

## 1. Introduction

Interruptions are ubiquitous in both our personal and professional lives. For example, studies show that interruptions cause one to lose between 5% (Gupta and Sharda, 2008) up to about 28% (Chen and Karahanna, 2014) of work time. Other studies found that it takes up to 25 min to resume the original activity (Mark et al., 2005; Addas and Pinsonneault, 2018b). Also, research indicates that about a quarter of all interrupted activities are not resumed at all (Addas and Pinsonneault, 2018b). Aside from affecting task performance (Gillie and Broadbent, 1989; Kapista and Blinnikova, 2003; Gluck et al., 2007; Basoglu et al., 2009; Mirhoseini et al., 2020), interruptions can also have different negative psychological consequences (Adamczyk and Bailey, 2004; Bailey and Konstan, 2006; Dodhia and Dismukes, 2009; Feldman and Greenway, 2021). Although research on interruption has steadily grown over time (Coiera, 2012; Puranik et al., 2020), the current state of research hampers a comprehensive overview of the concept of interruption, predominantly due to the fragmented nature of the existing literature. In fact, corresponding studies are published in outlets pertaining to various scientific disciplines such as Information Systems (IS), ergonomics, management, and psychology, among others (Janssen et al., 2015; Puranik et al., 2020).

Historically, the genesis of research on interruptions dates back to the 1920s with the experimental research and publications of Kurt T. Lewin. Based on field studies, Lewin and his students developed theories on human behavior as part of an experimental research program on action and effect psychology (Lewin, 1926). Blyuma W. Zeigarnik, a student in this experimental research program, examined the relationship between interruptions and memory. She found that individuals who are interrupted during tasks and are allowed to continue with other tasks recall the interrupted tasks more often than the uninterrupted ones. This phenomenon is referred to as the *Zeigarnik Effect* (for a comprehensive summary of the Zeigarnik Effect, please see Zeigarnik, 1927; Denmark, 2010). Another student in Lewin’s research program, Maria A. Rickers-Ovsiankina, investigated the resumption of interrupted actions. She identified a tendency to resume an interrupted action if it has not been completed. This phenomenon is referred to as the *Ovsiankina Effect* (Ovsiankina, 1928). Considering the findings of these pioneering studies, we conclude that interruptions may have significant psychological consequences, which may also affect consequences on other analytical levels. For example, memory effects (Zeigarnik, 1927) or resumption effects (Ovsiankina, 1928) may have significant organizational consequences, including those related to work satisfaction, performance, or productivity.

Research has produced many different perspectives and fundamental ideas over time. One of the foundational theories is the memory for goals theory by Altmann and Trafton (2002), which has been extensively used as the theoretical basis for the study of interruptions (for a detailed description of the memory for goals theory, please see Altmann and Trafton, 2002; Trafton et al., 2003). Following this theory, each task is associated with information which is stored in memory during interruptions and recalled after interruptions (Salvucci et al., 2009). Examining the anatomy of interruptions, with a focus on the characteristics, effects, and explanations of interruptions, provides helpful guidance for identifying the determinants of the disruptiveness of interruptions so that interrupted tasks can be successfully resumed after the interruption (Altmann and Trafton, 2002; Trafton and Monk, 2007). In this context, it should be noted that interference can occur in concurrent task execution when the ongoing task and the interruption require the same procedural resources (e.g., cognitive resources), as suggested by threaded cognition theory, another fundamental theory to consider in the study of interruptions, proposed by Salvucci and Taatgen (2008). As a result of research, however, various attributes of an interruption have emerged that allow interruptions to be distinguished from one another. For example, interruptions may be perceived as congruent or incongruent with a primary task (Brajnik and Gabrielli, 2010; Addas and Pinsonneault, 2018a), or as avoidable or unavoidable (Hillsden and Fenton, 2006; Hayes et al., 2015).

From an organizational perspective, the technological environment, particularly the information and communication technologies used in the professional environment (Hess et al., 2016), such as digital collaboration tools like Slack or Microsoft Teams, allow interruptions to be ubiquitous in the workplace (Wilkes et al., 2018) and thus can occur as planned or unplanned interruptions (Nystrom et al., 2010), thereby affecting whether a task is perceived as demanding or undemanding (Murray and Khan, 2014). Mobile technologies are particularly problematic in this regard, as interruptions often occur through audible and/or visual notifications (Tams et al., 2020), which have the potential to contribute to the development of addictive behavioral tendencies (e.g., looking at the smartphone every few minutes; Sha et al., 2019). Smartphones and other mobile technologies (e.g., wearables like smartwatches) also enable work-related leisure interruptions (e.g., work-related email while eating dinner at home) and leisure-related work interruptions (e.g., using private social media accounts during work hours) (Chen and Karahanna, 2014). Moreover, increasingly we observe the blurring of work and personal life, with companies increasingly expecting employees to be available outside of work hours (Ragsdale and Hoover, 2016). As a result, employees may fail to mentally separate themselves from work (Chen and Karahanna, 2018), which may lead to negative consequences such as work–family conflict (Wan et al., 2019), work-life conflict (Ragsdale and Hoover, 2016), and workplace exhaustion (Chen and Karahanna, 2018) as employees recovery processes are interrupted (Keller et al., 2020).

Given the long history of research related to interruptions, which started more than one hundred years ago with Lewin’s research program and corresponding contributions (e.g., Zeigarnik, 1927; Ovsiankina, 1928), and considering recent technological (e.g., smartphone), behavioral (e.g., users’ addictive behavioral tendencies), and organizational (e.g., expecting employees to be available outside of work hours) developments, one may assume that interruption science as an interdisciplinary academic field must already play an essential role in the scientific landscape. However, despite the growing interest in research on interruption, there is no comprehensive interdisciplinary overview of the concept of interruption. Considering both the economic and societal effects of interruptions, most of which are increasingly perceived as negative (Benlian, 2020; Salo et al., 2022), this finding of non-existence of such a fundamental overview is remarkable and also suggests that interruption science has not yet been established as an interdisciplinary research field in the scientific landscape. Against this background, the goal of the present paper is to develop a comprehensive interdisciplinary overview of the different attributes of an interruption. As it is impossible to develop a complete overview of a complex phenomenon like interruptions in one single paper, in this current article we focus on classifying interruptions. In short, in this paper we develop a taxonomy of interruptions.

To obtain a comprehensive overview of the concept of interruption, a scientific theory is essential as a starting point for research. One approach in scientific theory building is to analyze and describe a phenomenon of interest (Gregor, 2006) by identifying and classifying important attributes (McKelvey, 1982). This involves first identifying attributes and formally assigning them to recognized classes (McKelvey, 1978, 1982) by grouping objects according to their similarity (Bailey, 1994). McKelvey (1978, 1982) labels this approach as “science of diversity” since it examines commonalities while noting differences that distinguish groups from one another. Such a classification can therefore be a first step in obtaining a comprehensive overview of a research field. Specifically, it can provide a description of the field, as it offers the possibility to map it with its different components and relationships with the surrounding environment (McKelvey, 1978, 1982; Barki et al., 1988). Moreover, they reduce the complexity of the object of interest by identifying commonalities and differences among as well as within objects (McKelvey, 1982; Nickerson et al., 2013). Therefore, classifications can contribute to a better scientific understanding by introducing a common language for researchers and preventing the proliferation of synonyms (Barki et al., 1988).

However, there are several concepts for classifying objects of interest based on common characteristics (Bailey, 1994; Nickerson et al., 2013). To systematically analyze different interruption classifications, we used the classification concepts proposed by Bailey (1994) to distinguish classifications. We then structured and organized the interruption classifications using the methodological approach proposed by Nickerson et al. (2013) to develop a taxonomy of interruptions. Taxonomies as structuring artifacts greatly enhance understanding of complex phenomena (Szopinski et al., 2019). As an example, Torno et al. (2021) developed a taxonomy of mobile personal finance applications to encompass their dimensions and characteristics and their interrelated connections in terms of archetypes. Another example is the taxonomy of digital business models developed by Remane et al. (2017) to encompass the dimensions and characteristics of the personal mobility sector. Hence, we aim to develop a taxonomy of interruptions based on previous interruption classifications to provide a comprehensive interdisciplinary overview of the various attributes of an interruption as a foundation for the systematic investigation of interruptions.

Several literature reviews on interruptions exist. However, the identified related work comprises reviews that are either domain specific (O’Shea, 1999; Gurses and Xiao, 2006; Grundgeiger and Sanderson, 2009; James et al., 2009; Rivera-Rodriguez and Karsh, 2010; Li et al., 2012; Hayes et al., 2015) or focused on specific interruption attributes (Butterfield, 1964; Oshagbemi, 1995; van der Sijs et al., 2006; Trafton and Monk, 2007; Brajnik and Gabrielli, 2010; Turner et al., 2015; Stich, 2020; Wang et al., 2020). Table 1 outlines the identified reviews with the respective interruption emphasis. To the best of our knowledge, a comprehensive and systematic literature review on classifications of interruptions does not exist. Thus, the current article reviews previous interruption classifications to provide a comprehensive interdisciplinary overview of the concept of interruption. The goal of this paper is to expand the scope of the accumulated knowledge by developing a comprehensive perspective on the attributes of an interruption (Hart, 1988). Specifically, we address the following research question:

**Table 1:** Identified reviews on interruptions with their corresponding emphasis.

Review focus	Main interruption emphasis	Reference
**Interruption domains**	Interruptions and their adverse consequences in the domain of critical care and medication delivery	Grundgeiger and Sanderson (2009)
	Interruptions and their impact on design of information technology for information exchange and communication in the domain of healthcare	Gurses and Xiao (2006)
	Interruptions and their occurrence in the medication administration in a clinical setting in the domain of undergraduate nurse education	Hayes et al. (2015)
	Interruptions and their impact on the occurrence of dispensing errors in the domain of health care in community and hospital pharmacy	James et al. (2009)
	Interruptions and related task types and variables influencing the impact of interruptions in the domain of healthcare	Li et al. (2012)
	Interruptions and their impact on the occurrence of medication errors in the domain of clinical nurses	O’Shea (1999)
	Interruptions and their impact on safe and high-quality healthcare delivery in the domain of healthcare	Rivera-Rodriguez and Karsh (2010)
**Interruption attributes**	Interruption as an attribute for the impairment of the user experience on websites	Brajnik and Gabrielli (2010)
	Interruption as an attribute for a success-failure developmental conceptualization of task responses	Butterfield (1964)
	Interruption as an attribute to be addressed in managers’ time management strategies for coping with interruptions	Oshagbemi (1995)
	Interruption as an attribute of workplace stress experienced in virtual offices	Stich (2020)
	Interruption as an attribute for disruptiveness addressed in theoretical and applied research	Trafton and Monk (2007)
	Interruption as an attribute to be considered for developing intelligent interruption systems to assess the interruptibility of another person prior to an interaction	Turner et al. (2015)
	Interruption as an attribute to be considered in workflows for correct and effective processing of safety alerts	van der Sijs et al. (2006)
	Interruption as an attribute for work demands due to information and communication technologies in the workplace	Wang et al. (2020)

How can interruptions be classified within a taxonomy according to the current state of research?The remainder of this paper is structured as follows. Section 2 describes the review methodology consisting of a description of our literature search guided by the methodology for literature reviewing proposed by vom Brocke et al. (2009) to identify literature on the current state of research on interruption classifications. The subsections outline the definition of our review scope, the conceptualization of the review topic, and the literature search process. Section 3 then describes the structuring and organizing procedure of the identified literature on interruption classifications using the methodological approach for taxonomy development proposed by Nickerson et al. (2013). Section 4 follows with a presentation of the review results and a research agenda, including a discussion of the contributions and implications as well as avenues for future research. Finally, in Section 5, we provide a concluding statement.

## 2. Review methodology

In reviewing previous interruption classifications, we followed an established five-step methodology for literature reviewing proposed by vom Brocke et al. (2009). Specifically, the five steps of this methodology include:

1. Definition of the Review Scope
2. Conceptualization of the Review Topic
3. Presentation of the Process of the Literature Search
4. Execution of Literature Analysis and Synthesis
5. Presentation of a Research Agenda

Note that steps 4 and 5 are presented in the Results section as these steps refer to the review results (step 4) and their implications for future research (step 5).

### 2.1. Definition of the review scope

To systematically examine different interruption classifications, we applied the review methodology of vom Brocke et al. (2009), which draws upon Cooper (1988). According to Cooper’s taxonomy, six characteristics need to be considered when conducting a literature review. vom Brocke et al. (2009) point out that four characteristics can be combined independently (i.e., focus, goal, organization, and audience), while the other two are mutually exclusive (i.e., perspective and coverage). *First*, our systematic literature review focuses on research outcomes and applications of interruption classifications to provide an overview of the current state of research. *Second*, the goal of our systematic literature review is integration as it synthesizes interruption classifications proposed in previous research. *Third*, considering the history of the topic and based on the focus and goal of our systematic literature review, the purpose of collecting and summarizing literature is to demonstrate the value of interruption classifications through a neutral representation. *Fourth*, regarding the coverage, the goal of this paper is to capture all existing classifications on interruptions. Therefore, our review is exhaustive. *Fifth*, our review focuses on conceptual factors. *Sixth* and finally, we define the audience of our systematic literature review as general researchers. We summarize the characteristics of our review based on Cooper (1988) in Table 2, with the categories highlighted in gray being the focus of this review.

**Table 2:** Taxonomy of literature reviews (adapted from Cooper, 1988, p: 109).

**Characteristics**		**Categories**			
**(1)**	**Focus**	Research outcomes	Research methods	Theories	Applications
**(2)**	**Goal**	Integration	Criticism		Identification of central issues
**(3)**	**Perspective**	Neutral representation		Espousal of position	
**(4)**	**Coverage**	Exhaustive	Exhaustive and selective	Representative	Central or pivotal
**(5)**	**Organization**	Historical	Conceptual		Methodological
**(6)**	**Audience**	General researcher	Specialized researcher	Practitioners or policy makers	General public

### 2.2. Conceptualization of the review topic

As recommended by vom Brocke et al. (2009), we begin with a definition of the key term “interruption.” The starting point for our definition of interruption was recently published by Puranik et al. (2020), who synthesized research findings on work interruption to develop an integrative definition as a basis for future research. The authors’ analysis of prior definitions of work interruptions in 247 publications showed that interruptions have five attributes in common:

1. Suspension of an ongoing task’s execution
2. Unexpectedness of its occurrence
3. Presence of an interrupting task
4. Intention to resume the interrupted task
5. Interruption source is internal or external

Puranik et al. (2020) conclude that the attributes “*Suspension of an ongoing task’s execution*” and “*Unexpectedness of its occurrence*” are necessary elements of a definition of work interruption, while the other three attributes are irrelevant conditions from a definition perspective. As a result, Puranik et al. (2020) define work interruption as “*an unexpected suspension of the behavioral performance of, and/or attentional focus from, an ongoing work task*” (p: 817).

Research on interruptions is also constantly evolving over time. In recent years, for example, the phenomenon of interruptions caused by digital technologies, hereafter referred to as *IT-mediated interruptions*, has received increasing attention in practice and research. In this context, Couffe and Michael (2017) tellingly write that “interruptions of ongoing activities have spread since the development of and global increase in technology use and the general speeding in pace we all experience every day” (p: 163). Addas and Pinsonneault (2015) argue that particularly IT may trigger work interruptions as IT devices and programs interrupt office workers about 70 times per day during the completion of actual work tasks. Research also indicates that individuals are interrupted four to six times per work hour, frequently resulting in significant performance losses and productivity declines (Leroy, 2009; Galluch et al., 2015; Mirhoseini et al., 2020). This high number of IT-mediated interruptions during the workday is also a significant source of stress (Galluch et al., 2015; Stich et al., 2017). These exemplary research findings highlight the importance of knowledge for IT-mediated interruptions (Addas and Pinsonneault, 2015, 2018a,b; Galluch et al., 2015; Cheng et al., 2020; Feldman and Greenway, 2021). Against this background, and as an extension of Puranik et al.’s (2020) definition, Table 3 provides major definitions of the term “IT-mediated interruption.”

**Table 3:** Definitions of IT-mediated interruption.

Source	Definition
Addas and Pinsonneault (2015, p: 233)	“*IT-based external events with a range of content that captures cognitive attention and breaks the continuity of an individual’s primary task activities.* IT interruptions are a subset of work interruptions where technology creates the interruption (e.g., email; SMS; instant messaging).”
Addas and Pinsonneault (2018a, p: 1123)	“Temporary suspensions of an individual’s primary task activities to process information that is delivered by different media including face-to-face (F2F), telephone, and communication technologies (CT) such as email, texting, instant messaging, video conferencing, and social media.”
Chen and Karahanna (2018, p: 1025)	“a work-related occurrence via technology that impedes or delays an individual by breaking the continuity of an ongoing task”

### 2.3. Presentation of the process of the literature search

The starting point of our literature search consisted of three contributions on interruptions that focused on specific interruption attributes. For example, Addas and Pinsonneault examined how congruent and incongruent email interruptions affect individual task performance (Addas and Pinsonneault, 2018a) and developed a conceptual model to examine how communication technologies affect an individual’s work interruptions and how they affect group outcomes (Addas and Pinsonneault, 2018b). Chen and Karahanna (2018) investigated the consequences of technology-mediated work-related interruptions that occur during leisure time on work and nonwork outcomes. These three contributions provided an initial overview and seminal insights of the current state of research and the range of interruption attributes.

To identify further contributions on interruption classifications, we conducted a literature search based on existing recommendations for conducting literature searches (Webster and Watson, 2002; Kitchenham and Charters, 2007; vom Brocke et al., 2009). For our literature search, we used generic terms that represent interruption research (Break, Disruption, Distraction, Interruption, Intrusion, Suspension) and terms that represent concepts to classify interruptions (Classification, Concept, Conceptualization, Dichotomy, Division, Notation, Ontology, Organization, Systematics, Taxonomy, Terminology, Typology). Thereby, we excluded papers that did not classify interruptions or use distinctions between specific interruption types.

**Figure 1:**
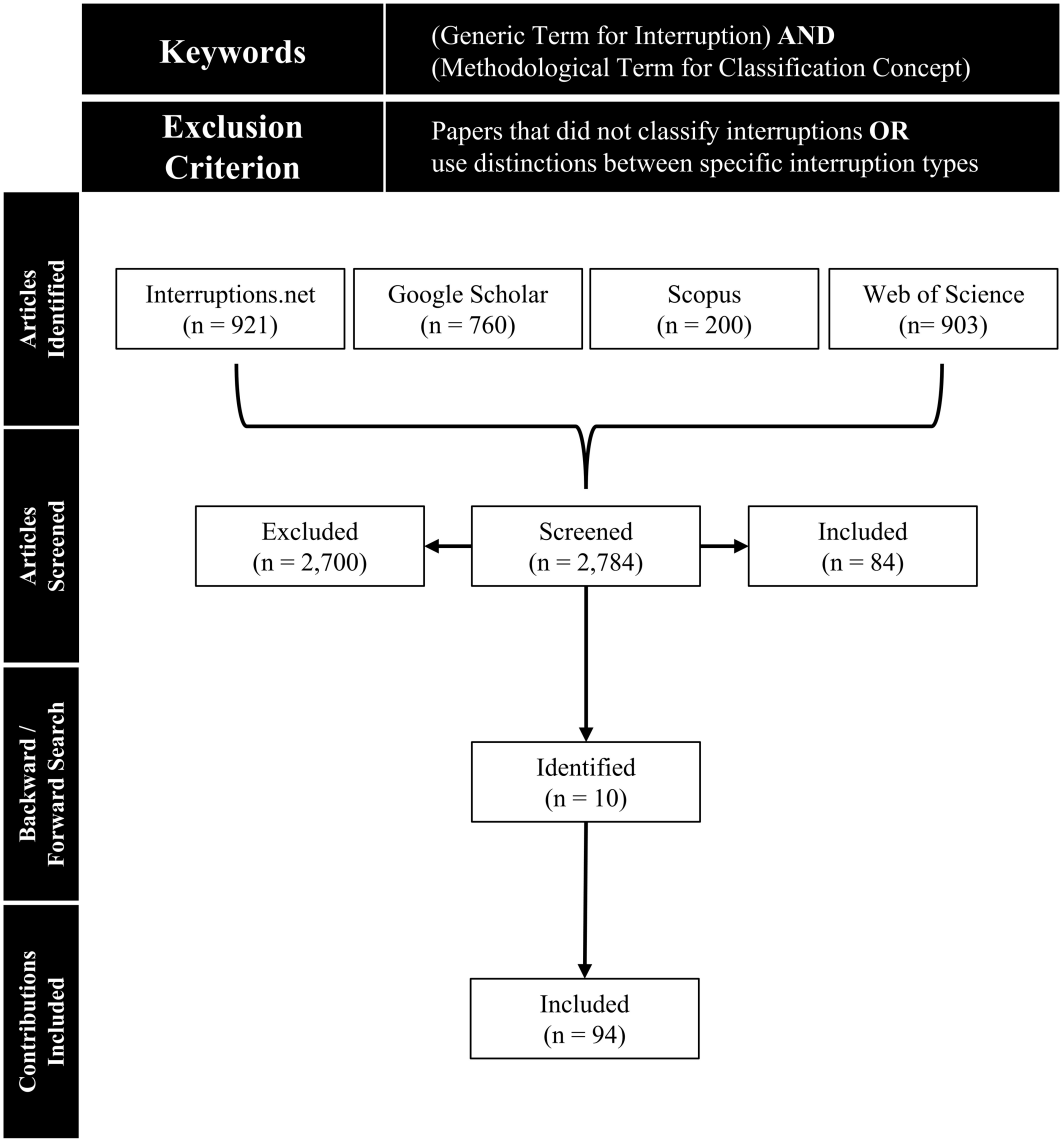
Flowchart of literature search process.

Our literature search initially started in a specialized database on interruptions^1^. In this source, it is not possible to constrain the search with the above keywords. Therefore, we manually reviewed all papers with respect to our research goal (i.e., systematic literature review on the classification of interruptions). In the next step, we searched literature in major academic databases (i.e., Google Scholar, Scopus, and Web of Science) using our keywords. Here, we limited the search to title and abstract, and reviewed the abstract and/or content of each paper to ensure that keywords were not merely cursory and that the contribution included content relevant to our research goal. In a final step, we conducted a backward and forward search to identify additional potentially relevant literature. Considering the long history of the research field, no publication year restriction was used for all searches, which supports the claim of this literature review to be exhaustive in coverage (Table 2). We only considered sources in English language. Figure 1 summarizes the literature search process.

This review covers literature on interruption classification published before and on April 22, 2022. In total, the literature base of our review comprises 94 contributions on interruption classifications, including 50 journal papers (53.2%), 36 conference proceedings papers (38.3%), 3 magazine articles (3.2%), 2 book chapter papers (2.1%), 2 reports (2.1%), and 1 dissertation (1.1%). Appendix A presents our literature base. The analysis of N = 94 publications allowed us to identify three different classification concepts, using the classification concepts proposed by Bailey (1994) to distinguish classifications. Out of the 94 papers, classifications were used in 78 papers (83%), typologies were used in 9 papers (9.6%), and taxonomies were used in 7 papers (7.4%). Appendix B lists the identified classification concepts.

## 3. Methodological approach to taxonomy development

For developing a taxonomy of interruptions, we followed the established five-step methodology for taxonomy development by Nickerson et al. (2013). Specifically, the five steps of this methodology include:

1. Definition of the Meta-Characteristic of the Taxonomy
2. Determination of the Ending Conditions in the Taxonomy Development
3. Selection of the Taxonomy Development Approach
4. Application of the Taxonomy Development Approach
5. Evaluation of the Taxonomy Stability

### 3.1. Definition of the meta-characteristic of the taxonomy

The first step of the taxonomy-building methodology from Nickerson et al. (2013) involves the definition of the meta-characteristic of the taxonomy. The meta-characteristic forms the foundation for the extraction of dimensions and specific characteristics of the later taxonomy. Moreover, it reflects the purpose and the expected benefit to users of the taxonomy at the highest level of abstraction.

The purpose of our systematic literature review is to integrate all existing interruption attributes by synthesizing the interruption classifications proposed in previous research. The expected benefit of the taxonomy is to provide an overview of the concept of interruption based on the current state of research, which also provides researchers with an important overview of the various attributes of an interruption. In reviewing the literature that classifies interruptions or provides different categories or types of interruptions, we were interested in two factors: the conceptual factor, which includes all conceptual dimensions of interruptions deductively derived from research related to interruptions, and the descriptive factor, which includes all perceived dimensions of interruptions inductively or intuitively derived from research related to interruptions. Specifically, our taxonomy aims to provide an overview of the various dimensions and characteristics of interruptions. In doing so, our taxonomy is intended to guide researchers as it summarizes different attributes of an interruption. Thus, the meta-characteristic of our taxonomy is high-level abstraction on the conceptual and descriptive structure of interruptions based on the current state of research.

### 3.2. Determination of the ending conditions in the taxonomy development

In the second step of the taxonomy-building methodology from Nickerson et al. (2013), the ending conditions for the taxonomy development process are determined. Ending conditions can be distinguished into eight objective and five subjective ending conditions, all of which we aim to satisfy at the end of the taxonomy’s development. In the final taxonomy, each dimension then contains characteristics that are mutually exclusive (i.e., no object has two different characteristics in one dimension) and collectively exhaustive (i.e., object has at least one characteristic in each dimension). Following Nickerson et al. (2013), the dimensions can be dichotomous or group multiple characteristics, depending on the objects’ information. Table 4 presents the ending conditions proposed by Nickerson et al. (2013).

**Table 4:** Ending conditions for taxonomy development (adapted from Nickerson et al., 2013, p: 344).

Ending condition type	Description
**Objective ending conditions**	All objects or a representative sample of objects have been examined
	No object was merged with a similar object or split into multiple objects
	At least one object is classified under every characteristic of every dimension
	No new dimensions or characteristics were added in the last iteration
	No dimensions or characteristics were merged or split in the last iteration
	Every dimension is unique and not repeated
	Every characteristic is unique within its dimension
	Each cell (combination of characteristics) is unique and is not repeated
**Subjective ending conditions**	Concise: Objects are limited without being unwieldy or overwhelming
	Robust: Objects provide enough differentiations among objects
	Comprehensive: All objects or a (random) sample of objects can be classified
	Extendable: New dimension or a new characteristic could easily be added in the future
	Explanatory: Dimensions and characteristics adequately explain the object

### 3.3. Selection of the taxonomy development approach

In this third step, the approach to be used for developing the taxonomy is selected according to the taxonomy-building methodology from Nickerson et al. (2013). Basically, two different approaches are possible: (1) the empirical-conceptual (E2C) approach, in which real objects are identified (e.g., through literature review) and then their dimensions and characteristics are grouped, and (2) the conceptual-empirical (C2E) approach, in which dimensions and characteristics are conceptualized independently of the real objects. For example, McFarlane (1997, 1998) selected an E2C approach to analyze the existing literature from various disciplines to identify dimensions and characteristics relevant to the design of user interfaces for human-computer interaction. The literature review allowed him to develop a classification of human interruption in the context of human-computer interaction, which he then used as a theoretical basis for further research (McFarlane, 1999, 2002; McFarlane and Latorella, 2002). Another example is Addas and Pinsonneault (2018a), who first selected a C2E approach by conceptualizing interruptions into congruent and incongruent email interruptions and then empirically investigated how these respective interruption types affect individual task performance. When developing taxonomies, one approach (e.g., Krug et al., 2012) or a combination of approaches (e.g., Geiger et al., 2011; Remane et al., 2017; Torno et al., 2021; Hengstler et al., 2022) may be selected. For the analysis and grouping of the objects we decided to select a combination to develop the taxonomy.

### 3.4. Application of the taxonomy development approach

The forth step of the taxonomy-building methodology from Nickerson et al. (2013) consists of applying the selected approach to taxonomy development. In this step it is necessary to determine common characteristics of the identified objects, which must be logical consequences of the meta-characteristic. In the resulting taxonomy, objects differ among themselves, which means that a characteristic cannot have the same value for multiple objects. For example, the work context of an interruption can be classified either with the characteristic during working hours or outside working hours (Chen and Karahanna, 2018), and not with both characteristics at the same time.

The decomposition of the 94 contributions into interruption classifications took four iterations. The final taxonomy contains 35 dimensions with unique characteristics for each dimension. In the following, we present our approach to taxonomy development by describing each iteration in more detail. Afterwards, in Table 5, we present our resulting taxonomy as a morphological box, which is a multidimensional matrix. The columns (i.e., dimensions) of the matrix show the conceptual structure of interruptions, and the rows (i.e., characteristics) provide the possible descriptive structure of interruptions based on the current state of research. All decisions regarding dimensions and characteristics during the taxonomy’s development were made collaboratively by the author team.

**Table 5:** Taxonomy of interruptions.

	Dimension	Characteristics					
Appearance Factors	D1: Cause	Human	Non-Human	System		Technology
	D2: Modality	Abstract Visual	Auditory	Auditory Icon		Earcon
		Heat	Kinesic	Light		Olfactory
		Paralinguistic	Pictographic	Speech		Signal
		Tactile	Textual	Verbal	Vibration	Visual
	D3: Retrieval Cue	Auditory Alert	Contextual Cue	Spatial Cue		Subtle Cue
		Visual Alert	Visual Cue		Visual Marker	
	D4: Transmission Channel	Face-to-Face	Mediated by Person	Mediated by System		Mediated by Technology
	D5: Type	Alarm	Alert	Call		Notification
		Reminder	Suggestion	Summon		Warning
Environmental Factors	D6: Domain	Work Interruption in Work Domain	Work Interruption in Private Domain	Private Interruption in Work Domain		Private Interruption in Private Domain
	D7: Expectation	Expected		Unexpected		
	D8: Persuasion	Persuasive		Unpersuasive		
	D9: Predictability	Predictable		Unpredictable		
	D10: Prevention	Avoidable		Unavoidable		
	D11: Work Context	During Working Hours		Outside Working Hours		
Task Factors	D12: Complexity	Demanding		Undemanding		
	D13: Coopetition	Cooperative		Competitive		
	D14: Frequency	Frequent		Infrequent		
	D15: Occurrence	During Simple Task		During Complex Task		
	D16: Origin	Within Current Task		External to Current Task		
	D17: Primary Task Relevance	Relevant		Irrelevant		
	D18: Primary Task Similarity	Similar		Dissimilar		
	D19: Purpose	Communication	Information	Task Outcome		Task Request
	D20: Relation	Task-related		Task-independent		
	D21: Temporal Lag	Short Lag		Long Lag		
	D22: Temporal Length	Short Duration		Long Duration		
	D23: Temporal Timing	Before Task Execution	During Task Execution		After Task Execution	
	D24: Urgency	Critical		Uncritical		
User Factors	D25: Cognitive Involvement	Low		High		
	D26: Coordination Method	Immediate	Mediated	Negotiated		Preemption	Scheduled	Sequential Processing	Simultaneity
	D27: Distractibility	Successful		Unsuccessful		
	D28: Event Trigger	Endogenous Event		Exogenous Event		
	D29: Information Availability	Available		Unavailable		
	D30: Response Level	Behavioral		Cognitive		
	D31: Stressor Controllability	Controllable		Uncontrollable		
Descriptive Factors	D32: Gender	Female	Male		Diverse	
		Inter	Open		No Information	
	D33: Intent	Break	Discrepancy		Distraction	
		Instruction	Intervention		Intrusion	
	D34: Interpersonal Relationship	Friendship	Power Relation		Work Roles	
	D35: Perspective	Interruption Initiator		Interruption Receiver		

**Iteration 1: E2C** – The first iteration aimed to identify all the characteristics of our 94 contributions to the classification of interruptions. The purpose of this initial analysis was to provide a valuable foundation for developing the dimensions of our taxonomy. During this process, we found that some of the 94 papers used the same interruption classification. For example, we identified 15 papers (i.e., Miyata and Norman, 1986; McFarlane, 1997, 1998; Mark et al., 2005; Brixey et al., 2007; Boehm-Davis and Remington, 2009; Jin and Dabbish, 2009; Clausen et al., 2010; Adler and Benbunan-Fich, 2013; McMurtry, 2014; Murray and Khan, 2014; Werner and Holden, 2015; Mentis et al., 2016; Couffe and Michael, 2017; Puranik et al., 2020) which classified interruptions as either internally triggered by an endogenous event (e.g., mind wandering) or externally triggered by an exogenous event (e.g., phone ringing). Moreover, we grouped papers describing the same interruption classification with different characteristics to encompass all different interruption characteristics. As an example, Licoppe (2010) distinguished between alarm, alert, call, summon, and warning as notification type of interruption, while Walji et al. (2004) distinguished between alert, notification, reminder, suggestion, and warning. We also grouped papers with the same interruption classifications, even if they were studied in a different context. For example, Federman (2019), Li et al. (2012) and Nystrom et al. (2010) distinguished between similar (interruption resembles the primary task) and dissimilar (interruption does not resemble the primary task) interruptions. Specifically, Federman (2019) focused on the similarity between the interruption task and primary task, while Li et al. (2012) investigated it during the execution of the primary task, and Nystrom et al. (2010) during the planning phase of a task. In total, we identified 39 unique interruption classifications, including 23 unidimensional classifications (59%) and 16 multidimensional classifications (41%). Appendix C provides an overview of the dimensionality of each interruption classification with the respective reference(s).

**Iteration 2: C2E** – In the second iteration, we attempted to develop an initial structure of our taxonomy of interruptions. To this end, we first analyzed the characteristics of multidimensional interruption classifications (see Table C2 in Appendix C) to develop dimensions according to their similarity. Indeed, each of these interruption classifications can be described by several characteristics. During this process, we found that some of the dimensions can be grouped together, which we considered the overarching structure of our taxonomy. For example, interruptions can appear in different types (e.g., Licoppe, 2010), use different modalities (e.g., Warnock et al., 2011a,b), and be transmitted through different channels (e.g., McFarlane, 1997, 1998). We have therefore grouped these dimensions and developed the overarching dimension “Appearance Factors.” In the same way, the overarching dimensions “Environmental Factors,” “Task Factors,” and “User Factors” were developed along with their corresponding characteristics.

**Iteration 3: C2E** – For the third iteration, we sought to complete the structure of our taxonomy of interruptions by incorporating the characteristics of the unidimensional interruption classifications (see Table C1 in Appendix C). To this end, we searched for appropriate connections between the characteristics of the unidimensional and multidimensional interruption classifications. As an example, in a multidimensional interruption classification, an interruption is distinguished according to content factors of a task such as complexity, relevance, and structure. Thereby, the unidimensional interruption classifications allowed us to develop appropriate dimensions with further specifications for complexity (i.e., the interruption is demanding or undemanding, depending on the difficulty of the interruption), relevance (i.e., the information is relevant or irrelevant to the main task), and structure (i.e., the interruption is similar or dissimilar to the main task), which we then assigned to the overarching dimension “Task Factors.” During this process, we also developed the overarching dimension “Descriptive Factors” when characteristics of unidimensional interruption classifications were applicable to multiple overarching dimensions of our taxonomy. One example is gender, as research shows that there are gender-specific differences in brain functioning (e.g., Riedl et al., 2010b). Thus, a different gender may cause the appearance of an interruption to be perceived differently or a resulting interruption task to be processed differently by the user. The development of this overarching dimension was therefore necessary to accommodate generic use cases within our taxonomy of interruptions.

**Iteration 4: E2C** – In the fourth iteration, we gradually included the remaining characteristics of the unidimensional interruption classifications to our taxonomy of interruptions. During this process, we did not need to develop additional dimensions as all remaining characteristics of the unidimensional interruption classifications could be classified and integrated into our existing taxonomy structure. This fourth iteration was thus also the last.

Finally, Table 5 presents our resulting taxonomy of interruptions in the form of a morphological box containing 35 dimensions with unique characteristics for each dimension that describe the conceptual and descriptive structure of interruptions based on the current state of research.

### 3.5. Evaluation of the taxonomy stability

In the fifth and final step of the taxonomy-building methodology from Nickerson et al. (2013), the resulting taxonomy needs to be evaluated regarding its usefulness to serve the intended purpose and intended users. This evaluation can be performed using different methods. For example, Torno et al. (2021) investigated the intended purpose by using the taxonomy to classify identified objects. Another example is Remane et al. (2017), who evaluated the usefulness of the developed taxonomy with intended users. Nickerson et al. (2013) indicate that such an evaluation of stability ensures the potential use of the developed taxonomy.

To evaluate the stability of our developed taxonomy of interruptions, our evaluation strategy involved a total of three evaluations: (1) evaluation of review validity, (2) evaluation of ending conditions, and (3) evaluation of taxonomy usefulness. Overall, this strategy allows us to evaluate the foundation, the process, and the outcome of taxonomy development by assessing its efficacy in classifying interruptions.

**Evaluation 1: Review Validity** – Validity refers to the degree to which a method (i.e., the design, the model, or the construct) measures what it purports to measure (Becker et al., 2013). Evaluating the validity of a planning process is essential, as it reveals whether or not solutions to problems can be found (Henderson and Sifonis, 1988). Therefore, the goal of this initial evaluation was to determine whether the foundation for the development of the taxonomy of interruptions was adequately established by the systematic literature review. To this end, instrument validation tests must be conducted to validate the research instruments (Straub, 1989). To validate our review methodology, we slightly modified the instrumental validity types of Becker et al. (2013) to evaluate potential validity threats related to our systematic literature review. This allowed us to identify four major validity concerns, which we were, however, able to mitigate accordingly in relation to our systematic literature review and its methodology.

1. **Descriptive Validity**: This validity type indicates the extent to which observations accurately reflect the phenomenon of interest. To mitigate this threat, we consider our approach to data collection to be exhaustive in coverage, as we have considered *all* papers that classify interruptions in a particular way or distinguish specific types of interruptions descriptively. The literature identified in this way is listed in Appendix A to objectify the data collection process. We have also designed a search process that allows us to continuously renew data collection.
2. **Theoretical Validity**: This validity type indicates the extent to which the true scope of a phenomenon of interest has been captured. To mitigate this threat, we carefully designed the search string by systematically combining an interruption term with a classification term and augmented the search with a forward and backward search. The identified papers were then analyzed collaboratively by the author team to avoid bias in data extraction and classification.
3. **Interpretive Validity**: This validity type indicates the extent to which the conclusions relate precisely to a phenomenon of interest. To mitigate this threat, we relied and drew conclusions on data obtained from our literature search.
4. **Repeatability**: This validity type indicates the extent to which the data of the research process are accurate and consistent when performed repeatedly. To mitigate this threat, we described the research process in detail, following an established methodology for literature reviews. We have also transparently presented all the data we received during the development of the taxonomy, such as our decomposition of the interruption classifications into unique interruption classifications in Appendix C, as Supplementary Material.

**Evaluation 2: Ending Conditions** – The taxonomy-building methodology from Nickerson et al. (2013) considers eight objective and five subjective end conditions to finalize the taxonomy development. Both types of ending conditions were satisfied at the end of the fourth iteration with our approach taxonomy development. We also ensured that our taxonomy of interruptions exhibits mutual exclusivity and collective exhaustiveness, since an interruption classification can be assigned to exactly one characteristic of the corresponding dimensions. Since the ending conditions were fully satisfied, the taxonomy development process was successfully terminated, which also contributes to the taxonomy’s stability.

**Evaluation 3: Taxonomy Usefulness** – Our final evaluation sought to explore the utility of the taxonomy we developed for its intended purpose and users. The intended purpose of our taxonomy was to provide an overview of the interruption classifications proposed in previous research as a foundation for research related to interruptions by identifying and classifying different attributes of an interruption. The intended users of our taxonomy are expected to be general researchers, who will be enabled by this high-level abstraction to grasp the conceptual and descriptive structure of interruptions based on the current state of research. Our taxonomy can therefore be used as a foundation for the discovery of additional interruption attributes or in its entirety, as well as for the systematic investigation of interruptions to further develop the taxonomy. While the former goal cannot be evaluated until new research on interruption is available, the taxonomy in Table 5 can be used as a heuristic to reveal new insights and opportunities for the latter objective, thereby indicating the usefulness of the developed taxonomy. Our recommendation here is to use the proposed taxonomy selectively and limit empirical research to specific dimensions or characteristics. Indeed, excessive use of the various dimensions and characteristics of the taxonomy may be too difficult for researchers to understand and apply to the systematic investigation of the concept of interruption. Accordingly, the taxonomy of interruptions could be examined from a variety of perspectives, including the following:

1. Interruptions can be mediated by different modalities. Similar to the studies by Warnock et al. (2011a, 2011b), future research could further investigate modalities and explore their effects on interruptions, such as the effects of specific modalities for various purposes (e.g., transmitting information) to improve human-computer interaction.
2. Interruptions can affect distinct domains at the interface between work and private life, which can be characterized with different attributes. Future research on work interruptions in the private domain may reveal additional attributes, as, for example, the non-work consequences of after-hours work-related technology, such as voluntary work-related technology use during non-work time (Derks et al., 2016; Ragsdale and Hoover, 2016; Schlachter et al., 2018), have hardly been investigated so far (Chen and Karahanna, 2018).
3. Interruptions can break the continuity of a task and have various implications for the execution of a running task. The timing of the interruption may be a determining factor in the impact of the interruption on the task (Federman, 2019). Qualitative methods (e.g., with interviews as a data collection method) might be appropriate to explore such findings. In doing so, our taxonomy could serve as categories for coding the collected data.
4. Interruptions can affect different areas of a user’s activity, such as how they coordinate interruptions (e.g., McFarlane and Latorella, 2002). Future experimental research may be appropriate to systematically investigate user behavior during interruptions by using distinct dimensions of our taxonomy, such as the role of cognitive involvement or temporal length in the interruption task. This could include combining various dimensions under specific conditions. In this regard, our taxonomy could serve as a guide to defining the interruption stimulus or the user’s response to the interruption.
5. Interruptions and their perception and processing can be distinguished by different descriptive factors, such as the interpersonal relationship between the interruption initiator and the interruption receiver (Harr and Kaptelinin, 2007). Future research could use the reporting dimensions and characteristics of our taxonomy to further explore the various attributes of interruptions and their interrelationships to gain a comprehensive overview of the concept of interruption.

## 4. Review results and research agenda

The presented taxonomy allows us to provide a comprehensive overview of the current state of research related to interruption attributes with their various dimensions and characteristics. Based on our results, in the following we describe:

1. Contributions and Implications of our results and
2. Avenues for Future Research.

### 4.1. Contributions and implications

We contribute to the interruption literature by providing a comprehensive overview of the conceptual and descriptive structure of interruptions based on the current state of research and deriving five overarching dimensions of interruption classification: “Appearance Factors,” “Environmental Factors,” “Task Factors,” “User Factors,” and “Descriptive Factors” that may be applicable to several of the above-mentioned factors. This result illustrates the complexity of interruptions by identifying the many factors that can play a role in an interruption. For example, the interruption stimulus may be mediated by different modalities (e.g., Warnock et al., 2011a, 2011b) that arise in a particular domain of the work-life interface (Chen and Karahanna, 2014). How then the user coordinates the interruption (e.g., McFarlane and Latorella, 2002) may depend on the complexity of the interruption task being processed (e.g., Federman, 2019) as well as the interpersonal relationship between the interruption initiator and the interruption receiver (Harr and Kaptelinin, 2007). Overall, the taxonomy of interruptions (see Table 5) represents a methodologically sound heuristic for the systematic investigation of interruptions, parts of which can also be used or further developed specifically for certain factors of interruptions.

With our research, we also extend related research on interruptions by providing an interdisciplinary overview of the various attributes of an interruption. Previous literature reviews investigated different interruption emphases, which were either limited to one domain or to specific interruption attributes (see Table 1). Our taxonomy also suggests that there may be various factors to be considered when examining interruptions that should not be treated separately. As an example, the interruption may be perceived as relevant to a primary task if the notification appears as an alarm, while the interruption may be perceived as irrelevant if the notification appears as a suggestion. Whether the interruption successfully or unsuccessfully captures the user’s attention could depend on, among other things, whether the interruption occurs during or outside working hours, whether the task to be completed is critical or uncritical, and also for whom in the organizational hierarchy the distracting task needs to be completed. We thus contribute to research on interruption with our taxonomy by providing a holistic view of interruptions and different factors to be considered.

Our taxonomy of interruptions was developed using an established methodology for literature reviews and is based on an established methodological approach for taxonomy development. As a result, it contains various dimensions and characteristics that provide a useful overview of the concept of interruption for further research. The main implication for research is that researchers can use our taxonomy as a foundation for the empirical research on interruptions. In this context, it is advisable to focus on specific factors since a single study cannot necessarily cover the entire spectrum of our taxonomy. However, various research strategies are possible. First, experimental research can be applied to test specific factors of our taxonomy. The focus could be limited to one dimension or a single characteristic. For example, Addas and Pinsonneault (2018a) investigated individual task performance of interruptions that were relevant and irrelevant to the primary task. Another example is Chen and Karahanna (2018), who examined the effects of interruptions caused by different types of technology. Second, qualitative research can be used to examine a particular factor and its possible interrelationship with other factors in more depth. Research has shown, among other things, that the timing of interruptions can have an impact on task performance. As an example, Murray and Khan (2014) analyzed time logs of daily work activities and found that interruptions have negative consequences on users’ task performance when they appear in the middle or at the end of the primary task, while interruptions at the beginning tend to have no effect. Also, Monk et al. (2004) revealed that interruptions in the middle of a task may be the most disruptive. In such a situation, employees would welcome the introduction of a new collaboration tool if it enabled them to avoid unexpected interruptions during task execution, as the perceived usefulness of such a change in the everyday work routine influences users’ resistance behavior (Laumer et al., 2016). Approaches to facilitate resumption of the main task after an interruption may include visual cues to the previous action (e.g., red cue; Trafton et al., 2005) or spatial cues to the primary task interface (e.g., partial view of the primary task surface; Ratwani et al., 2007). Notably, research also found positive effects of interruption, such as faster perceptual processing and fewer errors during task performance (e.g., Ratwani et al., 2006). A recent study by Nadj et al. (2023) even found that frequent interruptions with task-relevant information that contributes to the solution of the task have no negative impact on flow, a desirable state for task performance defined as “the holistic sensation that people feel when they act with total involvement” (Csíkszentmihályi, 1975, p: 36). Nonetheless, our taxonomy of interruptions could serve as a starting point for data collection. As an example, the taxonomy could be used as a basis for developing interview questions to explore general perceptions of work interruptions and management methods to deal with them (e.g., to explore context-specific dimensions and/or characteristics such as frequency or coordination methods of work interruptions; Stangl and Riedl, 2023). Our taxonomy could also serve as a guide for coding the collected data when applying qualitative research methods to identify constructs (e.g., to identify context-specific constructs such as types of work interruptions; Stangl and Riedl, 2023). Third, a mixed-methods approach can be applied, which combines elements and advantages of both quantitative and qualitative research approaches (Venkatesh et al., 2013, 2016). Indeed, the various features of mixed-methods research, through sequential qualitative and quantitative data collection and analysis, help to provide new and deeper insights into interruptions and expand our knowledge. Regardless of the research strategy chosen, our taxonomy can be used by researchers as a valuable foundation for a systematic investigation of interruptions.

From a practical perspective, our paper highlights the importance of the interdisciplinary academic research field of interruptions. Research on interruption continues to evolve as it is constantly confronted with new problems. As an example, IT-mediated interruptions are a growing problem that is increasingly becoming the focus of practice and research. In fact, the dynamics of digitalization can be seen, for example, in the technological environment, such as the information and communication technologies (ICT) that are used in the professional environment (Hess et al., 2016). Companies can achieve benefits in terms of productivity, effectiveness, and efficiency through the adoption and use of digital technologies (Melville et al., 2004). Such economic benefits from the use of digital technologies have also been demonstrated at the macroeconomic level (Ganju et al., 2016). However, besides improvements, ICT can also lead to negative consequences (Ayyagari et al., 2011; Galluch et al., 2015). For example, employees are expected to be more productive and work more efficiently because of the constant further development of the technologies used in the workplace in conjunction with increasing complexity. That this is not necessarily the case is shown by research results that demonstrate that greater strain on employees (i.e., IT-mediated interruptions) can lead to higher stress levels and negative effects on performance and productivity (for a review, please see Fischer and Riedl, 2017). Research has also shown, though, that training and practice can mitigate the disruptiveness of interruptions while improving primary task performance (Cades et al., 2006). As an implication for practice, research activities and findings from research on interruptions, such as our taxonomy of interruptions, are therefore particularly valuable. Indeed, the empirical study of our taxonomy is, in this respect, a way to provide society and businesses with new insights into interruptions.

To provide a foundation for systematic investigation of the concept of interruption, we developed a taxonomy of interruptions based on previous classifications of interruptions. The corresponding studies have been published in different scientific disciplines, which shows that there are various perspectives on interruptions. Due to the fragmented nature of the existing literature, however, it may be necessary to adapt or further specify certain dimensions or characteristics of our taxonomy for the research purpose. As an example, the “temporal length” dimension of our taxonomy classifies the duration of an interruption for a user as short or long. This distinction is based on the different lengths of interruptions used in experimental research to investigate cognitive effects of interruptions. For example, Hodgetts and Jones (2006) interrupted study participants for 6 or 18 s and Monk et al. (2008) interrupted for 3, 8, or 13 s during task performance to investigate attentional shift. Notably, the length of an interruption is a predictor that affects the disruptiveness of interruptions (Altmann and Trafton, 2002; Trafton and Monk, 2007). Other scientific disciplines, however, are also examining other cognitive effects of interruptions in this context, such as the neural mechanism of the brain during an interruption (Chen et al., 2021) or which parts of the human brain are associated with interruptions (Kalgotra et al., 2019). Hence, there are different perspectives on interruptions, which makes research on interruptions interdisciplinary and diverse in its research possibilities, both from a theoretical and practical perspective. The taxonomy of interruptions thereby provides a comprehensive interdisciplinary overview of the current state of research on interruption attributes with their various dimensions and characteristics, which also may contribute to better communication and mutual understanding of the concept of interruption between scientific disciplines and communities.

### 4.2. Avenues for future research

To guide future research on interruptions, we build on our results and derive five major avenues for future research. In the following, we present these avenues by describing each avenue in more detail.

**Avenue 1: Taxonomy Application** – One avenue for future research is the application of the taxonomy. Our taxonomy of interruptions identified five overarching dimensions of interruption classification based on the current state of research (see Table 5). However, due to the development and increasing use of technology and acceleration worldwide (Couffe and Michael, 2017), research on interruption is constantly facing new problems. Thereby, our taxonomy can be considered as a foundation to guide researchers in their research. Indeed, the underlying structure of our taxonomy, with its resulting dimensions and characteristics, allows for many different research opportunities with a variety of research strategies. Yet, depending on the research setting and context, it may be necessary to adapt the taxonomy to the requirements of the scholar. However, our taxonomy is useful for researchers to have an overview of the conceptual and descriptive structure of interruptions and the basic choices in planning a systematic investigation of interruptions. As an example, the taxonomy may serve as a foundation for qualitative research to code data. Future application of our taxonomy may also provide additional interruption dimensions and characteristics, along with an explanation of their interrelationship.

**Avenue 2: Taxonomy Extension** – The second avenue for future research is the advancement of the taxonomy. Our taxonomy abstracts the conceptual and descriptive structure of interruptions based on research at a high-level. An extended analysis of the dimensions and characteristics, though, may lead to further insights on the concept of interruption that have not yet been explored in research. For example, we identified one study in which the response to an interruption was classified at either the behavioral or cognitive level (Addas and Pinsonneault, 2015). However, research suggests that certain brain mechanisms are also involved in creating a genuine sense of agency to prepare for action (Haggard, 2017). The human thought process with the underlying conative, emotional, and motivational control of working memory, for example, determine whether information retained during an interruption is retained and stored in the short-term memory (Baddeley, 2003). In this context, the literature on Neuro-Information-Systems (NeuroIS) may serve as a starting point for further advancement of the taxonomy. NeuroIS is an interdisciplinary research field at the nexus of neurobiology and ICT within the IS discipline that uses neuroscience and neurophysiological tools and methods to better understand human cognition, emotion, and behavior in IS contexts (Riedl et al., 2010a, 2014, 2017; Riedl and Léger, 2016). NeuroIS studies typically collect data from neurophysiological measurements along with self-reported measures to investigate the use and effects of existing systems in more detail or to provide information for the development of new systems (Loos et al., 2010; Riedl et al., 2010a; Dimoka et al., 2012). This allows for a better understanding of the development, adoption, and impact of ICTs by examining the underlying human behavior and users’ cognitive and affective processes in human-computer interaction to determine how and why certain effects occur when using digital technologies (Riedl et al., 2010a; Dimoka et al., 2012; Riedl and Léger, 2016; Riedl et al., 2017). As an example, heart rate and heart rate variability are relevant measures for several IS research domains in the area of human-computer interaction, which are used to objectively measure a person’s ability to respond to environmental demands (Stangl and Riedl, 2022b). Such measures could be considered in a potential early warning system as physiological indicators to measure autonomic nervous system activity to measure stress-related disturbances (i.e., interruptions) during task performance (Stangl and Riedl, 2022a, 2022c). Indeed, empirical research showed that a combination of biometric data together with computer interaction data can predict with high accuracy the interruptibility of software developers at a given moment to avoid inappropriate moments for interruptions (Züger and Fritz, 2015; Züger et al., 2018). Another conceivable approach to extending the taxonomy is third-party evaluation. The evaluation of the usefulness of a taxonomy is necessary after the taxonomy development has been completed (Nickerson et al., 2013). Our evaluation strategy involved a multi-step process, examining the foundation, process, and outcome of taxonomy development by assessing its effectiveness in classifying interruptions. Conducting a qualitative study (e.g., interviews) with researchers who have expertise in research on interruptions could be an additional way to evaluate the usefulness of the taxonomy. This might lead to changes in the taxonomy or even open other, still hidden avenues. Nonetheless, further research to extend the taxonomy of interruptions seems promising and will lead to new avenues of research.

**Avenue 3: Taxonomy Investigation** – The third avenue for future research relates to empirical research on interruptions using our taxonomy as conceptual foundation. One possible avenue is to examine the determinants of the disruptiveness of interruptions using the dimensions and/or characteristics of our taxonomy. For example, an empirical study found that medical doctors perceive, on average, 10.58 interruptions per hour and nurses 11.65 interruptions per hour (Brixey et al., 2010), which is a higher number of interruptions than the average of 70 interruptions per day of an office worker (Addas and Pinsonneault, 2015). The taxonomy could thereby identify specific interruption attributes that also allow for comparison between similar or different target groups (e.g., the most frequent cause of interruptions). Another possible approach is to empirically examine dimensions or characteristics of the taxonomy in more detail. As an example, empirical research has examined the cognitive effects of temporally varying interruptions during task performance to explore attentional shifts (Hodgetts and Jones, 2006; Monk et al., 2008). To extend previous research findings, future research could, for example, incorporate neurophysiological measures to complement self-report or behavioral measures in research designs to draw more definitive conclusions about effects (e.g., Léger et al., 2014). From an organizational perspective, the interpersonal relationship between the interruption initiator and the interruption receiver (Harr and Kaptelinin, 2007), among other factors, might influence how interruptions are managed. To identify possible predictors that affect the disruptiveness of interruptions, such as the length of an interruption (Altmann and Trafton, 2002; Trafton and Monk, 2007), the overarching dimension “Descriptive Factors” or a combination of specific dimensions or characteristics within the taxonomy could be used as potential moderators. This would also lead to an extension of the taxonomy by exploring the interrelationships between the dimensions and characteristics in more detail. Nevertheless, since the taxonomy of interruptions contains 35 dimensions with unique characteristics for each dimension, it is more realistic for future research to selectively examine the proposed taxonomy and limit empirical research to specific dimensions or characteristics. As a starting point for developing possible hypotheses, researchers might also consider the literature on interruption classifications in general (see Appendix A) or the literature on the specific dimensionality of interruption classifications (see Appendix C). Overall, future research examining the anatomy of interruptions based on the comprehensive overview of the various attributes of an interruption provided by our taxonomy may provide helpful guidance for identifying additional determinants of the disruptiveness of interruptions and possible interrelationships among these factors.

**Avenue 4: Research Integration** – As a fourth avenue for future research, we emphasize the importance of research integration between different scientific disciplines and research fields. The scientific discourse on interruptions has been found in various disciplines. Indeed, research on interruptions is highly relevant for a variety of research disciplines, as evidenced by our literature base for deriving the taxonomy of interruptions. Among others, we found research from in IS (e.g., Addas and Pinsonneault, 2018a), medicine (e.g., Mamykina et al., 2017), or psychology (e.g., Couffe and Michael, 2017). However, we also found literature reviews that were limited to specific domains (see Table 1), although the results may be beneficial to several scientific disciplines. In line with Puranik et al. (2020), we characterize the current research on interruptions across the disciplines as scattered and insufficiently integrated. As evidence for this conclusion, we identified several papers which classified interruptions in the same way when we decomposed the interruption classification contributions (see Appendix C). We conclude that there is currently no cumulative research tradition on research on interruptions, notwithstanding that this interdisciplinary academic research field would benefit from a cumulative research tradition. Accordingly, further literature reviews could contribute to systematically mapping research output and targeting the potential of this research field, exemplified by this scoping review on interruption classifications (for an overview of the different literature review types, please see Paré et al., 2015; Schryen et al., 2017, 2020). As an example, to the best of our knowledge, a comprehensive and systematic literature review of the methods and measures used in research on interruptions does not yet exist. Such a methodology review, for example, advances a research field by providing perspectives on appropriate research methodologies and insights into the appropriate use of different methodologies (Pinsonneault and Kraemer, 1993). Thus, other comprehensive reviews that aim to integrate research across various scientific disciplines and research fields provide an important avenue for future research.

**Avenue 5: Paper Limitations** – The fifth and final avenue for future research is to address the limitations of our paper, which we will outline below. First, our taxonomy of interruptions draws on established methodologies for literature review and taxonomy development. However, the results of the taxonomy we developed, include some subjectivity in the design process during the conceptualization of the dimensions. We therefore note that our taxonomy is only one possible approach to interpreting the identified interruption classifications and that other developments of dimensions and characteristics may be possible. Second, our taxonomy provides a comprehensive perspective on classification of interruptions based on the current state of research that can guide researchers in future empirical studies. To this end, we reviewed papers published in a specialized database on interruptions and in the major academic databases Google Scholar, Scopus, and Web of Science. Searching additional databases (e.g., AIS eLibrary, ACM Digital Library, and IEEE Xplore), though, might reveal further dimensions and characteristics of interruptions. As an example, our literature search identified three papers that could also have been found using the IEEE Xplore database (i.e., van Solingen et al., 1998; Anhalt et al., 2001; Bolton et al., 2021). Therefore, it may be worthwhile for researchers to continue our systematic literature search in additional databases to potentially add additional dimensions or characteristics to our proposed taxonomy of interruptions. Third, our taxonomy provides a methodologically sound overview of the conceptual and descriptive structure of interruptions using an established methodology for literature reviews and an established methodological approach for taxonomy development. Since research on interruptions continues to face new problems over time and can therefore evolve rapidly, the results of our proposed taxonomy of interruptions are also to some extent transitory in nature. As such, it could be necessary to adjust the dimensions or characteristics to meet the new challenges posed by interruptions. Thereby, future desktop research replicating our original review methodology may reveal additional classifications of interruptions. Overall, addressing the above-mentioned limitations of our taxonomy is another highly promising avenue for future research on interruptions.

## 5. Concluding statement

The goal of this systematic literature review was to survey previous interruption classifications to propose a taxonomy of interruptions. During this process, we found that previous research on interruptions has already focused on several attributes. Our systematic literature review, though, complements this earlier research by providing a comprehensive review of the classification of interruptions as a unique contribution. By following an established methodology for literature review proposed by vom Brocke et al. (2009) and an established methodology for taxonomy development proposed by Nickerson et al. (2013), we were able to provide an overview of the various attributes of an interruption based on the current state of research. As a result, our taxonomy of interruptions contributes to research on interruptions by providing a comprehensive interdisciplinary overview of the conceptual and descriptive structure of interruptions.

Overall, our taxonomy of interruptions contributes as a foundation for the systematic investigation of interruptions, which also facilitates the establishment of interruption science as an interdisciplinary research field in the scientific landscape. Indeed, our taxonomy can guide researchers as a foundation for systematic investigation of interruptions. For example, researchers can apply our taxonomy to systematically examine identified dimensions and characteristics and explore their interrelationship. Researchers can also use the underlying structure of our taxonomy as a foundation for discovering additional interruption attributes, such as by further analyzing existing dimensions and characteristics, which further develops the taxonomy. Our taxonomy thus offers several promising avenues for further research in this interdisciplinary academic research field of interruptions. We therefore hope that our taxonomy of interruptions will encourage researchers to advance the scientific discourse on interruption with further contributions in both scientific research and practice.

## Data availability statement

The original contributions presented in the study are included in the article/Supplementary material, further inquiries can be directed to the corresponding author.

## Author contributions

RR was responsible for funding acquisition. FS and RR conceptualized the study and wrote the manuscript together. FS reviewed the literature under supervision of RR. All authors contributed to the article and approved the submitted version.

## Funding

This research was funded by the Austrian Science Fund (FWF) as part of the project “Technostress in Organizations” (project number: P 30865) and by the Austrian Research Promotion Agency (FFG) as part of the project “Interruption” at the University of Applied Sciences Upper Austria.

## Conflict of interest

The authors declare that the research was conducted in the absence of any commercial or financial relationships that could be construed as a potential conflict of interest.

## Publisher’s note

All claims expressed in this article are solely those of the authors and do not necessarily represent those of their affiliated organizations, or those of the publisher, the editors and the reviewers. Any product that may be evaluated in this article, or claim that may be made by its manufacturer, is not guaranteed or endorsed by the publisher.

## Footnotes

^1^https://interruptions.net/literature.htm (last access on April 22, 2022).
