# Supplementary material for: Interruption science as a research field: Towards a taxonomy of interruptions as a foundation for the field
Source: Front Psychol. 2023 Mar 22;14:1043426. doi: 10.3389/fpsyg.2023.1043426 (PMC10074991; doi:10.3389/fpsyg.2023.1043426)
Supplement: Supplementary file 1 [file Data_Sheet_1.docx]

Supplementary Material

Interruption Science as a Research Field: Towards a Taxonomy of Interruptions as a Foundation for the Field

Fabian J. Stangl* and René Riedl

*** Correspondence:** Fabian J. Stangl: Fabian.Stangl@fh-steyr.at

# Appendix A: Literature on Interruption Classifications

This appendix presents the literature base of our systematic literature review. The keywords used were generic terms that represent interruption research and terms that represent concepts to classifying interruptions. The databases included a specialized database on interruptions and the major academic databases Google Scholar, Scopus, and Web of Science. Our literature search identified 94 papers on interruption classification, presented in the **Table A1**, published before and on April 22, 2022.

Table A1. Overview of Literature Base

| **Reference** | **Title** | **Outlet Type** |
| --- | --- | --- |
| Addas and Pinsonneault (2015) | *The many faces of information technology interruptions: A taxonomy and preliminary investigation of their performance effects* | Journal |
| Addas and Pinsonneault (2018a) | *E-mail interruptions and individual performance: Is there a silver lining?* | Journal |
| Adler and Benbunan-Fich (2013) | *Self-interruptions in discretionary multitasking* | Journal |
| Altmann and Trafton (2004) | *Task interruption: Resumption lag and the role of cues* | Conference |
| Altmann and Trafton (2015) | *Brief lags in interrupted sequential performance: Evaluating a model and model evaluation method* | Journal |
| Altmann et al. (2014) | *Momentary interruptions can derail the train of thought* | Journal |
| Andrews et al. (2009) | *The effect of alert type to an interruption on primary task resumption* | Conference |
| Anhalt et al. (2001) | *Toward context-aware computing: Experiences and lessons* | Magazine |
| Arroyo and Selker (2003) | *Arbitrating multimodal outputs: Using ambient displays as interruptions* | Conference |
| Boehm-Davis and Remington (2009) | *Reducing the disruptive effects of interruption: A cognitive framework for analysing the costs and benefits of intervention strategies* | Journal |
| Bolton et al. (2021) | *Detecting interruption events using EEG* | Conference |
| Brajnik and Gabrielli (2010) | *A review of online advertising effects on the user experience* | Journal |
| Brixey et al. (2004) | *Proposing a taxonomy and model of interruption* | Conference |
| Brixey et al. (2007) | *A concept analysis of the phenomenon interruption* | Journal |
| Brudzinski et al. (2007) | *Goal and spatial memory following interruption* | Conference |
| Cades et al. (2007) | *Does the difficulty of an interruption affect our ability to resume?* | Conference |
| Chen and Karahanna (2014) | *Boundaryless technology: Understanding the effects of technology-mediated interruptions across the boundaries between work and personal life* | Journal |
| Chen and Karahanna (2018) | *Life interrupted: The effects of technology-mediated work interruptions on work and nonwork outcomes* | Journal |
| Cheng et al. (2020) | *Investigating the impact of IT-mediated information interruption on emotional exhaustion in the workplace* | Journal |
| Chewar and McCrickard (2003) | *Educating novice developers of notification systems: Targeting user-goals with a conceptual framework* | Conference |
| Chewar et al. (2004) | *Unpacking critical parameters for interface design: Evaluating notification systems with the IRC framework* | Conference |
| Clausen et al. (2010) | *Disruption management in the airline industry—Concepts, models and methods* | Journal |
| Cohen (1980) | *Aftereffects of stress on human performance and social behavior: A review of research and theory* | Journal |
| Couffe and Michael (2017) | *Failures due to interruptions or distractions: A review and a new framework* | Journal |
| Cutrell et al. (2001) | *Notification, disruption, and memory: Effects of messaging interruptions on memory and performance* | Conference |
| Czerwinski et al. (1991) | *Interruptions in multitasking situations: The effects of similarity and warning* | Report |
| Czerwinski et al. (2000) | *Instant messaging: Effects of relevance and time* | Conference |
| Darmoul et al. (2013) | *Handling disruptions in manufacturing systems: An immune perspective* | Journal |
| Edwards and Gronlund (1998) | *Task interruption and its effects on memory* | Journal |
| Fabian et al. (2004) | *Designing the claims reuse library: Validating classification methods for notification systems* | Conference |
| Federman (2019) | *Interruptions in online training and their effects on learning* | Journal |
| Gievska and Sibert (2004a) | *A framework for context-sensitive coordination of human interruptions in human-computer interaction* | Conference |
| Gievska and Sibert (2004b) | *Empirical validation of a computer-mediated coordination of interruption* | Conference |
| Gievska and Sibert (2005) | *Using task context variables for selecting the best timing for interrupting users* | Conference |
| Gievska et al. (2005) | *Examining the qualitative gains of mediating human interruptions during HCI* | Conference |
| Gillie and Broadbent (1989) | *What makes interruptions disruptive? A study of length, similarity, and complexity* | Journal |
| Grandhi and Jones (2010) | *Technology-mediated interruption management* | Journal |
| Harr and Kaptelinin (2007) | *Unpacking the social dimension of external interruptions* | Conference |
| Hayes et al. (2015) | *Medication errors in hospitals: A literature review of disruptions to nursing practice during medication administration* | Journal |
| Hillsden and Fenton (2006) | *Improving practice and patient safety through a medication systems review* | Journal |
| Hodgetts and Jones (2006) | *Interruption of the Tower of London task: Support for a goal-activation approach* | Journal |
| Jett and George (2003) | *Work interrupted: A closer look at the role of interruptions in organizational life* | Journal |
| Jin and Dabbish (2009) | *Self-Interruption on the computer: A typology of discretionary task interleaving* | Conference |
| Kern et al. (2004) | *A model for human interruptability: Experimental evaluation and automatic estimation from wearable sensors* | Conference |
| Kirmeyer (1988) | *Coping with competing demands: Interruptions and the Type A pattern* | Journal |
| Li (2001) | *Cooperative and intrusive interruptions in inter- and intracultural dyadic discourse* | Journal |
| Li et al. (2004) | *Reconceptualizing interruptions in physician-patient interviews: Cooperative and intrusive* | Journal |
| Li et al. (2005) | *Interruption and involvement in discourse: Can intercultural interlocutors be trained?* | Journal |
| Li et al. (2012) | *A systematic review of the psychological literature on interruption and its patient safety implications* | Journal |
| Licoppe (2010) | *The ‘crisis of the summons’: A transformation in the pragmatics of ‘notifications’, from phone rings to instant messaging* | Journal |
| Lin et al. (2013) | *Don’t interrupt me! An examination of the relationship between intrusions at work and employee strain* | Journal |
| Magrabi et al. (2010) | *Why is it so difficult to measure the effects of interruptions in healthcare?* | Conference |
| Mamykina et al. (2017) | *Driven to distraction: The nature and apparent purpose of interruptions in critical care and implications for HIT* | Journal |
| Mark et al. (2005) | *No task left behind? Examining the nature of fragmented work* | Conference |
| McCrickard and Chewar (2003) | *Attuning notification design to user goals and attention costs* | Magazine |
| McCrickard et al. (2003) | *A model for notification systems evaluation—Assessing user goals for multitasking activity* | Journal |
| McFarlane and Latorella (2002) | *The scope and importance of human interruption in human-computer interaction design* | Journal |
| McFarlane (1997) | *Interruption of people in human-computer interaction: A general unifying definition of human interruption and taxonomy* | Report |
| McFarlane (1998) | *Interruption of people in human-computer interaction* | Dissertation |
| McFarlane (1999) | *Coordinating the interruption of people in human-computer interaction* | Conference |
| McFarlane (2002) | *Comparison of four primary methods for coordinating the interruption of people in human-computer interaction* | Journal |
| McMurtry (2014) | *Managing email overload in the workplace* | Journal |
| Mentis et al. (2016) | *A systematic review of the effect of distraction on surgeon performance: Directions for operating room policy and surgical training* | Journal |
| Miyata and Norman (1986) | *Psychological issues in support of multiple activities* | Book Chapter |
| Monk (2004) | *The effect of frequent versus infrequent interruptions on primary task resumption* | Conference |
| Monk et al. (2002) | *The attentional costs of interrupting task performance at various stages* | Conference |
| Monk et al. (2004) | *Very brief interruptions result in resumption cost* | Conference |
| Monk et al. (2008) | *The effect of interruption duration and demand on resuming suspended goals* | Journal |
| Murray and Khan (2014) | *Impact of interruptions on white collar workers* | Journal |
| Nystrom et al. (2010) | *The effect of interruption similarity in planning tasks* | Conference |
| Ortiz de Guinea and Webster (2013) | *An investigation of information systems use patterns: Technological events as triggers, the effect of time, and consequences for performance* | Journal |
| Oulasvirta and Saariluoma (2004) | *Long-term working memory and interrupting messages in human–computer interaction* | Journal |
| Puranik et al. (2020) | *Pardon the interruption: An integrative review and future research agenda for research on work interruptions* | Journal |
| Ratwani and Trafton (2008) | *Spatial memory guides task resumption* | Journal |
| Ratwani et al. (2007) | *Using peripheral processing and spatial memory to facilitate task resumption* | Conference |
| Ratwani et al. (2008) | *Predicting postcompletion errors using eye movements* | Conference |
| Renaud (2000) | *Expediting rapid recovery from interruptions by providing a visualization of application activity* | Conference |
| Speier et al. (1999) | *The influence of task interruption on individual decision making: An information overload perspective* | Journal |
| Sproull (1984) | *The nature of managerial attention* | Book Chapter |
| Stich (2020) | *A review of workplace stress in the virtual office* | Journal |
| Sykes (2011) | *Interruptions in the workplace: A case study to reduce their effects* | Journal |
| Tiedge (1975) | *Clarifying the concept of distraction* | Journal |
| Trafton et al. (2003) | *Preparing to resume an interrupted task: Effects of prospective goal encoding and retrospective rehearsal* | Journal |
| Trafton et al. (2005) | *Huh, what was I doing? How people use environmental cues after an interruption* | Conference |
| van Solingen et al. (1998) | *Interrupts: Just a minute never is* | Magazine |
| Walji, Brixey, et al. (2004) | *A theoretical framework to understand and engineer persuasive interruptions* | Conference |
| Walji, Johnson-Throop, et al. (2004) | *The case for persuasive interruptions in healthcare* | Conference |
| Wang et al. (2020) | *How does the use of information communication technology affect individuals? A work design perspective* | Journal |
| Warnock et al. (2011a) | *The impact of unwanted multimodal notifications* | Conference |
| Warnock et al. (2011b) | *The role of modality in notification performance* | Conference |
| Werner and Holden (2015) | *Interruptions in the wild: Development of a sociotechnical systems model of interruptions in the emergency department through a systematic review* | Journal |
| Yang (1996) | *Interruptions and intonation* | Conference |
| Yang (2001) | *Visualizing spoken discourse: Prosodic form and discourse functions of interruptions* | Conference |
| Zijlstra et al. (1999) | *Temporal factors in mental work: Effects of interrupted activities* | Journal |

# Appendix B: Concepts of Interruption Classifications

This appendix lists all 94 publications on interruption classifications in terms of their classification concept. For this purpose, we used the classification concepts proposed by Bailey (1994) to distinguish classifications, which allowed us to categorize the classifications in the publications into three classification concepts. A brief description of each classification concept follows before **Table B1** lists all 94 publications by their classification concept with the corresponding reference.

**Classification Concept 1: Classification** – This classification concept is defined as the general process or result of grouping entities by similarity, which can be either unidimensional (i.e., a single dimension or characteristic) or multidimensional (i.e., several dimensions or characteristics). As an example, Boehm-Davis and Remington (2009) classified interruptions as triggered either internally by an endogenous event (e.g., mind wandering) or externally by an exogenous event (e.g., phone ringing). The process of grouping entities by similarity can be conceptual or empirical.

**Classification Concept 2: Typology** – Compared to classifications, this classification concept is multidimensional (i.e., several dimensions or characteristics) and based on a theoretical ideal or model. Typologies are thus more complex than classifications. For example, Chen and Karahanna (2014) classified interruptions according to the interrupting and interrupted domains, allowing four different types of interruptions to be distinguished: a) Interruptions that originate from and occur in the work domain; b) Interruptions that originate from and occur in the personal life domain; c) Interruptions that originate from the work domain but occur in the personal life domain; and d) Interruptions that originate from the personal life domain but occur in the work domain. The process of grouping entities by similarity can be done conceptually with a theoretical foundation or can be inferred by deduction. This classification concept does not necessarily rely on empirical data, though such data may be used for verification purposes toward the end of the grouping process. For example, Federman (2019) proposed a typology that identifies temporal, content, and urgency characteristics as the three main conceptual dimensions for distinguishing between different interruption types and their effects on learning. To validate the proposed typology, she conducted an online survey to assess these dimensions, which was able to provide support for the typology. Another example is the research of Gievska and Sibert (2004a), who identify task context, user context, and environmental context as the three main conceptual dimensions relevant to the process of coordinating human interruptions. They investigated the scope and utility of their proposed theoretical model through an exploratory user study and a laboratory study (Gievska and Sibert, 2004a, 2004b, 2005; Gievska et al. 2005).

**Classification Concept 3: Taxonomy** – Taxonomy as a classification concept is often used as a synonym of classification for the general process or the result of grouping entities according to their similarity. However, the term taxonomy should be reserved for the empirical or inductive process of grouping entities by similarity, which distinguishes it from the conceptual or deductive grouping process of a typology. Exceptions to this usually involve the subsequent identification of empirical cases for conceptual typologies. McFarlane (1997, 1998), for example, analyzed existing literature from several different disciplines relevant to the design of user interfaces for human-computer interaction. As a result, he developed a classification of human interruption in the context of human-computer interaction as a theoretical basis for research, which he termed the "Taxonomy of Human Interruption". Notably, this conceptual classification has only been partially validated for the different coordination methods for user performance in human-computer interaction (McFarlane, 1997, 1998, 1999, 2002; McFarlane and Latorella, 2002). A taxonomy can also be based on a constructed type related to empirical research. As an example, Brixey et al. (2004) conducted a systematic literature review on previously published studies on interruptions in the healthcare and developed a taxonomy to classify interruptions related to technology acceptance or adoption (e.g., introduction of a new communication technology) in the clinical setting.

Table B1. Overview of Interruption Classification Concepts

| **Classification Concept** | **References** |
| --- | --- |
| **Classification** | Addas and Pinsonneault (2018a); Adler and Benbunan-Fich (2013); Altmann and Trafton (2004, 2015); Altmann et al. (2014); Andrews et al. (2009); Arroyo and Selker (2003); Boehm-Davis and Remington (2009); Bolton et al. (2021); Brajnik and Gabrielli (2010); Brixey et al. (2007); Brudzinski et al. (2007); Cades et al. (2007); Chen and Karahanna (2018); Cheng et al. (2020); Chewar and McCrickard (2003); Chewar et al. (2004); Clausen et al. (2010); Cohen (1980); Couffe and Michael (2017); Cutrell et al. (2001); Czerwinski et al. (1991, 2000); Darmoul et al. (2013); Edwards and Gronlund (1998); Fabian et al. (2004); Gillie and Broadbent (1989); Grandhi and Jones (2010); Harr and Kaptelinin (2007); Hayes et al. (2015); Hillsden and Fenton (2006); Hodgetts and Jones (2006); Jin and Dabbish (2009); Kern et al. (2004); Kirmeyer (1988); Li (2001); Li et al. (2004, 2005, 2012); Licoppe (2010); Magrabi et al. (2010); Mamykina et al. (2017); Mark et al. (2005); McCrickard and Chewar (2003); McCrickard et al. (2003); McMurtry (2014); Mentis et al. (2016); Miyata and Norman (1986); Monk (2004); Monk et al. (2002, 2004, 2008); Murray and Khan (2014); Nystrom et al. (2010); Ortiz de Guinea and Webster (2013); Oulasvirta and Saariluoma (2004); Puranik et al. (2020); Ratwani and Trafton (2008); Ratwani et al. (2007, 2008); Renaud (2000); Speier et al. (1999); Sproull (1984); Stich (2020); Sykes (2011); Tiedge (1975); Trafton et al. (2005, 2003); van Solingen et al. (1998); Walji, Brixey, et al. (2004); Walji, Johnson-Throop, et al. (2004); Wang et al. (2020); Warnock et al. (2011a, 2011b); Werner and Holden (2015); Yang (1996, 2001); Zijlstra et al. (1999) |
| **Typology** | Anhalt et al. (2001); Chen and Karahanna (2014); Federman (2019); Gievska and Sibert (2004a, 2004b, 2005); Gievska et al. (2005); Jett and George (2003); Lin et al. (2013) |
| **Taxonomy** | Addas and Pinsonneault (2015); Brixey et al. (2004); McFarlane (1997, 1998, 1999, 2002); McFarlane and Latorella (2002) |

# Appendix C: Dimensionality of Interruption Classifications

This appendix provides an overview of the dimensionality of each interruption classification across all 94 publications on interruption classifications. For this purpose, we distinguished the classification concepts into unidimensional and multidimensional classifications, as proposed by Bailey (1994), to differentiate each interruption classification. While the former dimensionality is based on a single dimension or characteristic, the latter is based on several dimensions or characteristics. **Table C1** provides an overview of the unidimensional interruption classifications, while **Table C2** provides an overview of the multidimensional interruption classifications with their respective reference(s).

Table C1. Overview of Unidimensional Interruption Classifications

| **Description** | **Reference(s)** |
| --- | --- |
| Actionable (interruption requires response) vs. informational (interruption requires one-way dissemination of information) interruption content | Addas and Pinsonneault (2015); Anhalt et al. (2001) |
| Avoidable vs. unavoidable interruption | Hayes et al. (2015); Hillsden and Fenton (2006) |
| Behavioral (interruption triggers action) vs. cognitive (interruption triggers cognition) interruption processing | Addas and Pinsonneault (2015) |
| Congruent (information that is relevant to primary task) vs. incongruent (information that is not relevant to primary task) interruption | Addas and Pinsonneault (2015, 2018a); Bolton et al. (2021); Brajnik and Gabrielli (2010); Cheng et al. (2020); Chewar and McCrickard (2003); Chewar et al. (2004); Czerwinski et al. (2000); Fabian et al. (2004); McCrickard and Chewar (2003); McCrickard et al. (2003) |
| Controllable vs. uncontrollable interruption | Cohen (1980) |
| Cooperative (interruption is supportive) vs. competitive (interruption is not supportive) interruption | Yang (1996, 2001) |
| Demanding task interruption (task with high difficulty level) vs. undemanding task interruption (task with low difficulty level) | Federman (2019); Murray and Khan (2014) |
| During working hours interruption vs. outside working hours interruption | Chen and Karahanna (2018) |
| Expected vs. unexpected interruption | Brixey et al. (2007); Darmoul et al. (2013); Ortiz de Guinea and Webster (2013) |
| Frequent (interruptions occur often during task execution) vs. infrequent (interruptions occur rarely during task execution) interruption | Monk (2004); Speier et al. (1999); Zijlstra et al. (1999) |
| High-rated (interruption is critical) vs. low-rated interruption (interruption is uncritical) | Chewar and McCrickard (2003); Chewar et al. (2004); Fabian et al. (2004); McCrickard and Chewar (2003); McCrickard et al. (2003) |
| Human initiated interruption vs. by a non-human entity-initiated interruption | Brixey et al. (2004); Mamykina et al. (2017); McFarlane (1997, 1998) |
| Internally initiated interruption by an endogenous event (e.g., mind wandering) vs. externally initiated interruption by an exogenous event (e.g., phone ringing) | Adler and Benbunan-Fich (2013); Boehm-Davis and Remington (2009); Brixey et al. (2007); Clausen et al. (2010); Couffe and Michael (2017); Jin and Dabbish (2009); Mark et al. (2005); McFarlane (1997, 1998); McMurtry (2014); Mentis et al. (2016); Miyata and Norman (1986); Murray and Khan (2014); Puranik et al. (2020); Werner and Holden (2015) |
| Interruption initiator vs interruption receiver | Brixey et al. (2004) |
| Persuasive (interruption that convinces the individual) vs. unpersuasive (interruption that does not convince the individual) interruption | Walji, Johnson-Throop, et al. (2004) |
| Planned (scheduled) vs. unplanned (unscheduled) interruption | Brixey et al. (2007) |
| Predictable vs. unpredictable interruption | Cohen (1980) |
| Similar (interruption resembles the primary task) vs. dissimilar (interruption does not resemble the primary task) interruption | Czerwinski et al. (1991); Edwards and Gronlund (1998); Federman (2019); Gillie and Broadbent (1989); Li et al. (2012); Magrabi et al. (2010); Nystrom et al. (2010); Oulasvirta and Saariluoma (2004); Speier et al. (1999) |
| Successful (interruption causes a premature end of an activity) vs. unsuccessful (interruption does not cause a premature end of an activity) interruption | Li (2001); Li et al. (2004, 2005) |
| System-generated vs. mediated by the system interruption | Addas and Pinsonneault (2015) |
| Task-related interruption vs. task-independent interruption | Cutrell et al. (2001); Sykes (2011) |
| Technology-mediated vs. non-technology-mediated interruption | Grandhi and Jones (2010); McFarlane (1997, 1998); Stich (2020); Wang et al. (2020) |
| Within running application interruption (e.g., application reports a problem) vs. another external application interruption (e.g., computer needs to be updated to install updates for another application) | Renaud (2000) |

Table C2. Overview of Multidimensional Interruption Classifications

| **Description** | **Reference(s)** |
| --- | --- |
| By cognitive involvement of the interruption (e.g., attention unit, change in attention, distractibility) | Brixey et al. (2004); Sproull (1984) |
| By content factor of the interruption (e.g., complexity, relevance, structure) | Addas and Pinsonneault (2015); Anhalt et al. (2001); Federman (2019); Gillie and Broadbent (1989); Speier et al. (1999) |
| By conveyance channel of the interruption (e.g., face-to-face, mediated by a machine, mediated by a person, mediated by a system, meditated by other animate object, other direct communication channel) | Addas and Pinsonneault (2015); McFarlane (1997, 1998) |
| By coordination method of the interruption (e.g., immediate, mediated, negotiated, preemption, scheduled, sequential processing, simultaneity) | Brixey et al. (2004); Cheng et al. (2020); Kirmeyer (1988); Magrabi et al. (2010); McFarlane (1997, 1998, 1999, 2002); McFarlane and Latorella (2002) |
| By domain of the interruption (e.g., interruptions that originate from and occur in the work domain, interruptions that originate from and occur in the personal life domain, interruptions that originate from the work domain but occur in the personal life domain, interruptions that originate from the personal life domain but occur in the work domain) | Chen and Karahanna (2014) |
| By environmental context of the interruption (e.g., availability of information, context, expectation, location, social constraints, situation awareness, socio-technical factors) | Brixey et al. (2004); Gievska and Sibert (2004a, 2004b, 2005); Gievska et al. (2005); Magrabi et al. (2010) |
| By individual characteristic of a person receiving the interruption (e.g., gender, goals, interpersonal relation, relative context to interruption source, state of desired satisfaction state, state/limitations of personal resource) | Gievska and Sibert (2004a, 2004b, 2005); Gievska et al. (2005); Harr and Kaptelinin (2007); Kern et al. (2004); McFarlane (1997, 1998) |
| By modality of the interruption (e.g., abstract visual, auditory, auditory icon, earcon, heat, kinesic, light, olfactory, paralinguistic, pictographic, speech, signal, tactile, textual, verbal, vibration, visual) | Arroyo and Selker (2003); Brixey et al. (2004); Magrabi et al. (2010); McFarlane (1997, 1998); van Solingen et al. (1998); Warnock et al. (2011a, 2011b) |
| By notification type of the interruption (e.g., alarm, alert, call, notification, reminder, suggestion, summon, warning) | Licoppe (2010); Walji, Brixey, et al. (2004) |
| By reason to interrupt (e.g., communication, expected outcome, perform another task, provide information, other purpose) | Anhalt et al. (2001); Brixey et al. (2004); Magrabi et al. (2010); Mamykina et al. (2017); McFarlane (1997, 1998) |
| By retrieving cue when interrupted (e.g., auditory alert, contextual cue, spatial cue, subtle cue, visual alert, visual cue, visual marker) | Altmann and Trafton (2004); Andrews et al. (2009); Brudzinski et al. (2007); Cutrell et al. (2001); Czerwinski et al. (2000); Ratwani et al. (2007, 2008); Trafton et al. (2005) |
| By task context of the interruption (e.g., communicative intent, expected performance outcome, number of ongoing tasks, task complexity, task criticality, task dependencies, task difficulty, task type) | Cades et al. (2007); Czerwinski et al. (2000); Gievska and Sibert (2004a, 2004b, 2005); Gievska et al. (2005); Magrabi et al. (2010); Monk et al. (2002) |
| By technology transmission type of the interruption (e.g., pager, telephone) | Brixey et al. (2004) |
| By temporal factor of the interruption (e.g., temporal frequency, temporal lag, temporal length, temporal timing) | Altmann and Trafton (2015); Altmann et al. (2014); Anhalt et al. (2001); Brixey et al. (2004); Czerwinski et al. (2000); Federman (2019); Gillie and Broadbent (1989); Hodgetts and Jones (2006); Magrabi et al. (2010); Monk et al. (2002, 2004, 2008); Ratwani and Trafton (2008); Trafton et al. (2003) |
| By type of work interruption (e.g., breaks, discrepancy, distraction, instruction, intervention, intrusion) | Addas and Pinsonneault (2015); Jett and George (2003); Lin et al. (2013); Tiedge (1975) |
| By urgency of the interruption (e.g., importance of interruption, status of interruption initiator, transmission of interruption) | Federman (2019) |
